# The effect of human amniotic epithelial cell on dendritic cell differentiation of peripheral blood monocytes: An experimental study

**DOI:** 10.18502/ijrm.v13i6.7286

**Published:** 2020-06-30

**Authors:** Bahare Keshavarzi, Meraj Tabatabaei, Amir Hasan Zarnani, Fahime Ramezani Tehrani, Mahmood Bozorgmehr, Nariman Mosaffa

**Affiliations:** ^1^Department of Immunology, School of Medicine, Shahid Beheshti University of Medical Sciences, Tehran, Iran.; ^2^Nanobiotechnology Research Center, Avicenna Research Institute, ACECR, Tehran, Iran.; ^3^Reproductive Endocrinology Research Center, Research Institute for Endocrine Sciences, Shahid Beheshti University of Medical Sciences, Tehran, Iran.

**Keywords:** Amniotic membrane, Dendritic cells, Human placenta, Immunomodulation, Monocyte.

## Abstract

**Background:**

The amniotic membrane plays an important role in maintaining a healthy pregnancy. The main population cells from amniotic membrane include human amnion epithelial cells (hAECs) which have been shown to possess immunomodulatory properties.

**Objective:**

The proximity of hAECs with monocyte leads to the generation of tollerogenic dendritic cells.

**Materials and Methods:**

hAECs were obtained from normal pregnancy. Peripheral blood monocytes were isolated by anti-CD14 MACS method. Co-cultures of monocytes and hAECs were established in Transwell chambers supplemented with granulocyte-macrophage colony-stimulating factor (GM-CSF) and interleukin-4 (IL-4) in the absence and presence of lipopolysaccharide (LPS) to produce immature and mature DCs, respectively. Immunophenotyping of the obtained DCs was done through flow cytometry and the production of cytokines was measured by ELISA. Mixed leukocyte Reaction (MLR) was also performed for the functional assessment of DCs.

**Results:**

Immunophenotyping of [hAECs - Immature DC (iDC)] and [hAECs - iDC] + LPS cells revealed that the expression of CD1a, CD80, CD86, CD40, HLA-DR, and CD83 markers showed no significant difference as compared with the control group (iDCs and mDCs alone). In the [hAECs-iDCs] + LPS cells, the percentage of CD14 cells at the ratio of 1:2.5 showed significant differences compared to the control group. The production of IL-10 and IL-12 showed no significant difference in any of the cultures as compared to the control groups. Also, co-cultured DCs did not inhibit proliferation of lymphocyte.

**Conclusion:**

Our findings show that factors secreted from cultured hAECs are unable to generate of tollerogenic dendritic cells. To achieve a better understanding of other mechanisms more investigations are needed.

## 1. Introduction

During pregnancy human embryo is surrounded by amnion and chorion membranes. The amnion is in direct contact with the fetus and plays an important role in the process of normal pregnancy (1). The properties of the amniotic membrane, such as its low immunogenicity (2), anti-inflammatory (3), anti-scarring and wound healing (4) effects, bring out a wide range of applications for this membrane in regenerative medicine. Two types of cells possessing different extents of plasticity have been isolated from the amniotic membrane and described by subsequent investigations, including human amnion epithelial cells (hAECs) and human amnion mesenchymal stromal cells (hAMSCs) (5). Feto-maternal immune tolerance plays an important role in normal pregnancy. This immune tolerance has motivated many investigators to study the immune properties of the human amniotic cells. Previous studies have indicated that hAECs exhibit low levels of immunogenicity; consequently, these cells could be transplanted onto the rabbit cornea without any clinical side effects or tissue rejection (6). The lack of human leukocyte antigen (HLA)-II and co-stimulatory molecules in the presence or absence of interferon gamma (IFN-γ), low expression of HLA-I (5, 7), and constitutive expression of HLA-G (8) by hAECs may be partly responsible. Another important feature of hAECs is their immunomodulatory properties. hAECs produce migration inhibitory factors and inhibit the migration of macrophages (2).

It has been indicated that prostaglandin E2 (PGE2) and transforming growth factor beta (TGF-β1) are the most powerful immunomodulatory factors produced by hAECs, which have been also shown to suppress T cell proliferation (9). Besides, hAECs inhibit proliferation of peripheral blood mononuclear cells (PBMCs) previously activated by mitogens (7, 10). Moreover, the secretary factors from hAECs have been shown to induce apoptosis of activated T cells (2). Due to the immunomodulatory properties of hAECs, these cells are expected to be potentially used as a therapy to modulate pathogenic immune responses. Recently, investigations indicated that the hAECs can be used for the treatment of inflammation-mediated spinal cord injury (11), hepatic fibrosis (12), multiple sclerosis (9), and inflammatory pulmonary fibrosis (13).

The basic mechanisms of the immunosuppressive properties of hAECs are not yet clear. Dendritic cells (DCs) Known as the most potent professional antigen-presenting cells for naive T cells and are important role in initiating and regulating immune responses (14). The maturation state of DCs in combination with environmental and endogenous factors determine stimulatory or tolerogenic properties of lymphocytes (15). The absence of costimulatory molecules and major histocompatibility complex class II (MHCII) on tolerogenic DCs lead to induce T cell anergy, preventing T cell activation.

Many protocols have been established to generate tolerogenic DCs using numerous agents, including glucocorticoids such as dexamethasone, mycophenolic acid, vitamin D3, retinoic acid, and the combination of dexamethasone and vitamin D3, all of which prevent the maturation of the dendritic cell. Therefore, ex-vivo-generated tolerogenic DCs as therapeutic vaccines are suitable candidates to “re-establish antigen-specific tolerance in autoimmune disorders” (16). Recent investigations indicate that mesenchymal stem cells (MSCs) obtained from various tissues can affect the maturation of DCs and inhibit DC differentiation of peripheral blood monocytes (16-19). Since the findings on the immunomodulatory properties of hAECs are in accordance with those explained for MSCs obtained from the amnion membrane, bone marrow (20), cord blood (20) and adipose tissue (21), so far.

In the current research, we studied whether secreted factor from hAECS can also inhibit monocytes` differentiation towards DCs. Furthermore, we investigated the production of interleukin (IL)-10 and IL-12 by DCs and the ability of these DCs to stimulate T cell proliferation.

## 2. Materials and Methods

### Reagents and antibodies

Reagents used in the cell culture and Ficoll-Paque 1077 were obtained from Gibco (UK). Fluorescein isothiocyanate (FITC)-conjugated monoclonal antibodies (mAb) against human cytokeratin, CD34, CD9, CD38, HLA-G, HLA-DR and phycoerythrin (PE)-conjugated mAbs against CD133, CD29, CD44, SSEA-4, CD73, CD10, HLA-I, and corresponding isotype controls were all purchased from BD biosciences (USA). PE-conjugated anti-CD105 and unconjugated anti-STRO-1 mAb were from R&D System (USA). Polyclonal rabbit anti-human OCT-4 and FITC-conjugated goat anti-rabbit antibodies were purchased from Abcam (USA) and Sigma (USA), respectively. FITC-conjugated sheep anti-mouse Ig was obtained from Avicenna Research Institute (Iran). Anti-CD14-coated microbeads and magnetic-activated cell sorting (MACS) separation columns were purchased from Miltenyi Biotech (Germany). PE-conjugated CD14 antibody was obtained from BD (USA). FITC-conjugated anti-CD80, HLA-DR, PE-conjugated anti-CD14, CD83, CD86, CD1a, and PECY5-conjugated anti CD40 and corresponding isotype controls were all purchased from BD (USA). Recombinant IL-4 and granulocyte-macrophage colony-stimulating factor (GM-CSF) were obtained from R&D (USA). Lipopolysaccharide (LPS) was purchased from Sigma (USA). IL-10 and IL-12 ELISA kits were obtained from R&D (USA). [3H] thymidine was purchased from Amersham (USA). Glass-fiber filter was obtained from Titertek (USA). Human Regulatory T Cell Staining Kit was obtained from eBiosciences (USA).

The sampling of human Buffy coats from healthy donors and early term placental units of healthy mothers due to the inclusion criteria was randomized selection.

The sampling of human Buffy coats from healthy donors and early term placental units of healthy mothers due to the inclusion criteria was randomized selection.

### Isolation of human amniotic epithelial cells 

Human placentas were obtained from uncomplicated term pregnancies delivered by elective Cesarean section from healthy women aged 20 to 35 yr. Amniotic epithelial cells were isolated and characterized using flow cytometry in accordance with the protocols described by Tabatabaei and colleagues (22). In brief, amniotic membranes were peeled free from the underlying chorion and washed several times. The membranes were divided into four parts and were placed in cell culture flask of 75 cm${}^{2}$. Then, 15-20 mL of buffer containing 0.05% trypsin-EDTA and 20 µg/ml DNase was added to each flask to perform four successive digestion steps. The viability of harvested cells was examined by trypan blue exclusion test. Single cell suspensions obtained from steps 2-4 were pooled and analyzed by flow cytometry for the expression of cytokeratin, CD9, CD10, CD29, CD73, CD34, CD38, CD44, CD105, CD133, HLA-I, HLA DR, HLA-G, SSEA-4, STRO-1, and OCT-4.

### Cultivation of hAECs

Harvested hAECs from each membrane were cultured in complete tissue culture medium Roswell park memorial institute (RPMI 1640) + FBS10% + Penicillin/Streptomycin in 25 cm${}^{2}$ tissue culture flasks. Two days after that, semi-adherent cells were removed to be used for co-culture.

### Isolation and purification of monocytes from human peripheral blood

Blood samples were obtained from healthy donors admitted to the Blood Transfusion Organization, Tehran, Iran according to the policy approved by the Ethical Committee. PBMCs were isolated by Ficoll-Paque 1077 density gradient centrifugation. Peripheral blood monocytes were isolated by anti-CD14-coated microbeads and MACS separation columns through positive selection according to the manufacturer's protocol. Monocytes were stained with PE-conjugated anti-CD14 antibody. The flow cytometry analysis confirmed a purity of > 98%.

### Induction of monocyte-derived DCs

Based on previous studies, peripheral blood monocytes were differentiated to iDCs by the use of IL-4 and GM-CSF. mDCs were developed by adding LPS in iDCs culture. In order to developing iDCs, The monocytes (1 × 10${}^{6}$/ml) were cultured in 1 ml RPMI complete medium (supplemented with 10% fetal bovine serum (FBS), recombinant GM-CSF (50 ng/ml) and IL-4 (100 ng/ml)) and seeded in 24-well culture plate at 1 ml/well and followed by incubation in 37°C for 3-5 days. Then iDCs were stimulated by adding 100 ng/ml LPS and incubated for 2 more days in order to developing mDCs.

### Co-culture of monocyte-derived DCs and hAECs

Co-cultures of monocytes and hAECs (at passages 0) were established in Transwell 0.4 µm pore size membrane chambers obtained from SPL (Korea). Monocytes were co-cultured with hAECs at monocytes: hAEC ratios of 1:1, 1:2.5, and 1:10. For the production of immature DCs (iDCs), 1.5 × 10${}^{6}$ monocytes were cultured in the inferior chambers of the Transwell plates and hAEC were cultured in the insert chambers at a total volume of 2.5 mL of complete medium (RPMI + 10% FBS, 0.2 M L-glutamine, non-essential amino acids, 1% Sodium pyruvate) with GM-CSF (100 ng/mL) and IL-4 (50 ng/mL) for five days. The cells from this co-culture were named [hAECs-iDCs]. For the production of mature DCs (mDCs) from the co-cultures, the same approach was followed with the only difference that on the fifth day, the supernatant (1 mL) of the monocytes were collected and replaced with fresh medium containing LPS (50 ng/mL). The supernatant (1 mL) of the hAECs were collected as well and replaced with fresh medium and the cells were cultured for two more days. The cells from these co-cultures were named [hAECs-iDCs] + LPS. In both co-culture models, monocytes alone were used as the control groups.

### Flow cytometry analysis

For the immunophenotyping of the DCs obtained from our co-cultures, anti-CD80, HLA-DR (FITC-conjugated), anti-CD83, CD14, CD86 and CD1a (PE-conjugated), anti-CD40 (PECY5-conjugated) antibodies were used. In all the tests, the isotype-matched antibodies were used as negative controls. Briefly, the cell suspensions were incubated for 30 min at 4°C in a staining solution (PBS + 2% FBS + antibody). After the incubation, the cells were washed and analyzed by flow cytometry (Partec, Germany).

### Cytokine assays

To evaluate the production of IL-12 and IL-10, co-culture supernatants were collected on day 5 for iDC and day 7 for mDC and stored at -80°C until being tested. [hAECs-iDCs] and [hAECs-iDCs] + LPS co-culture supernatants were used for the test groups and iDC, mDC, and hAECs alone were used as the control groups. Measurement of Cytokineswere assayed with an ELISA Kit according to the manufacturer's instructions. “The optical density of the wells plate were read using Anthos ELISA reader at 570 and 450 nm (as reference wavelength).The minimal detection limits for IL-10 and IL-12 was 31.25 pg/mL”.

### Proliferation assay

#### Preparation of Peripheral blood lymphocytes (PBLs) as responder cells in the mixed leukocyte reaction (MLR)

In order to prepare PBLs, appropriate blood volumes were taken from healthy donors and PBMCs were isolated using standard procedures with Ficoll-isopaque and Percoll density gradient centrifugation. Next, the harvested cells were cultured in complete tissue culture medium in T25 (SPL, Korea) tissue culture flasks for 2 hr in a 37°C incubator supplemented with 5% CO${}_{2}$. Then, the non-adherent cells, that is, the PBLs were slowly collected and after washing at 200 g for 5 min in fresh medium, the PBLs were used as responders for the allogeneic mixed lymphocyte reaction (allo-MLR).

### Mixed leukocyte reaction 

MLR was performed to assess the function of cells within the [hACEs-iDCs] + LPS group. Briefly, [hACEs-iDCs] + LPS cells were cultured with PBL at the three effector: target ratios of 1:1, 1:2.5, and 1:10 for five days. All cultures were carried out in triplicate in round-bottom 96-well tissue culture plates, at a final volume of 200 µL of RPMI complete medium. Co-culture of [hAECs-iDCs] + LPS cells with PBLs were deemed as the test groups and the co-culture of mDC with PBLs were deemed as our controls. [hAECs - iDCs] + LPS cells alone and PBLs alone were used as negative controls, and the co-cultures of Phytohemagglutinin (PHA) with PBLs were used as positive controls. T-cell proliferation was assessed after five days by adding [3H] thymidine (1 µCi/well) for 16-18 hr. Cells were then harvested with a cell harvester on the glass- fiber filter. Then the filters were placed onto the scintillation vials and the scintillation liquid was added, and finally, thymidine incorporation was measured using a β-Counter (LKB Wallac, 1410, USA).

### Regulatory T cell assay

In order to evaluate the ability of [hAECs-iDCs] + LPS cells to generate T-regs, [hAECs-iDCs] + LPS and mDC cells were co-cultured with PBLs for five days at a ratio of 1:1. PBLs alone were used as negative controls. Cells were stained with the Human Regulatory T Cell Staining Kit and anti-CD4, CD25, and Foxp3 antibodies and analyzed by flow cytometry (Partec, Germany).

### Ethical consideration

This study was approved by the ethical committee of Shahid Beheshti University of Medical Sciences, Tehran, Iran (code: 92556) and all participants signed a written consent form before enrolment in the study.

### Statistical analysis

The statistical analysis was performed using GraphPad Prism 5.0 (GraphPad Software, Inc., La Jolla, CA, http://www.graphpad.com) software. One-way ANOVA was used to compare the means of normally distributed data, and nonparametric Kruskal-Wallis, Mann-Whitney, and Dunnett tests were used for the not normally distributed data. The results were presented as mean ± SD; p < 0.05 was considered statistically significant. The results are representative of four individual experiments.

## 3. Results

### Isolation and immunophenotyping of hAECs 

**Figure 1:**
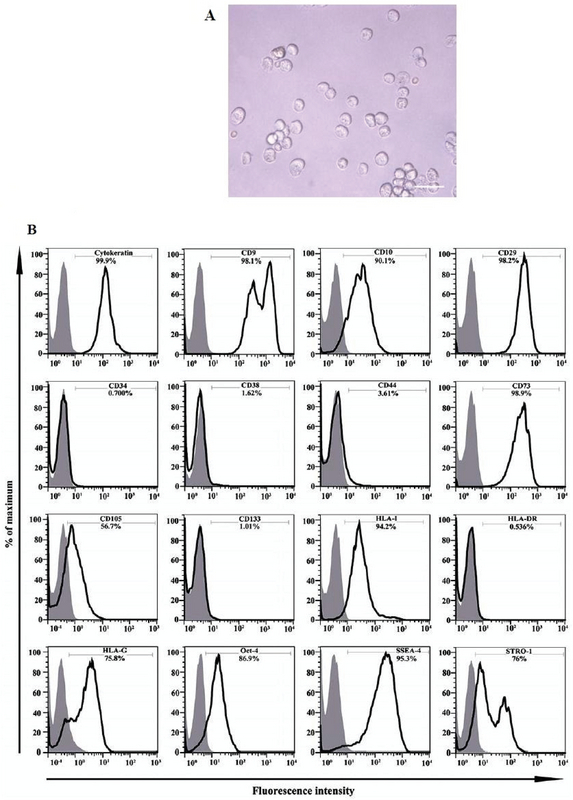
(A) Morphology of freshly isolated human amniotic epithelial cells (hAECs)(×100). (B) Immunophenotype of isolated hAECs Open histograms display cells stained with monoclonal antibodies and filled histograms display cells stained with isotype-matched control antibodies. The percentage of positive cells is shown in each plot. The results are representation of six separate experiments.

About 70-120 × 106 per/membrane hAECs were isolated from each placenta unit with high purity as determine by the evaluation of cytokeratin expression (≥ 90%). Trypan blue dye exclusion test disclose a viability of > 95%. These cells appeared as flat round cells (Figure 1A). Immunophenotyping of hAECs was evaluated by flow cytometry. Accordingly, hAECs were positive for cytokeratin, CD9, CD10, CD29, CD73, CD105, HLA-I, HLA-G, STRO-1, SSEA-4, and OCT-4 while negative for HLA-II, CD34, CD38, CD44, and CD133 (Figure 1B).

### Immunophenotyping of monocyte and monocyte-derived cells in the absence of hAEC

**Figure 2:**
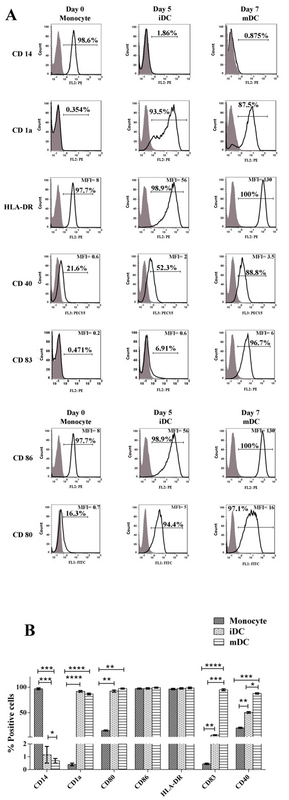
Immunophenotyping of monocyte and monocyte-derived cells in the absence of Human amniotic epithelial cells. (A) Immunophenotype profile of monocytes (Day 0), immature dendritic cells (day 5) and mature dendritic cells (day 7). Monocytes were isolated by magnetic activated cell sorting and were differentiated to immature dendritic cells s by the use of IL-4 and GM-CSF for five days. Then, immature dendritic cells were stimulated by adding 100 ng/ml LPS and incubated for two more days (day 7). Immunophenotype of cells were measured using flow cytometry. Open histograms display cells stained with monoclonal antibodies and filled histograms display cells stained with isotype-matched control antibodies. experiments. (B) The figure displays the percentages of cells staining positive for each marker; * indicates statistically significant differences of cells. iDC: immature dendritic cells, mDC: mature dendritic cells.

A analysis of CD14 expression levels by flow cytometry showed that monocytes were isolated with a purity of 98 ± 0.9%. As expected, purified monocytes were positive for the CD86 and HLA-DR and negative for CD80, CD83, CD40, and CD1a. Immunophenotyping of iDC on day 5 showed that during the process of monocyte differentiation into iDC, CD14 strongly reduced and CD1 (specific marker of monocyte-derived DC) significantly increased as a result of differentiation. Also, these cells were positive for CD80, CD86, and CD40. Immunophenotyping of mDC on day 7 showed that during the process of iDC differentiation into mDC, CD1a and CD83 increased significantly. These mDC cells were negative for CD14 and positive for CD83, CD80, CD86, CD40, and HLA-DR. These results demonstrate the successful differentiation of iDC to mDC (Figure 2).

### Immunophenotyping of monocyte derived iDC cells in the presence of hAEC

**Figure 3:**
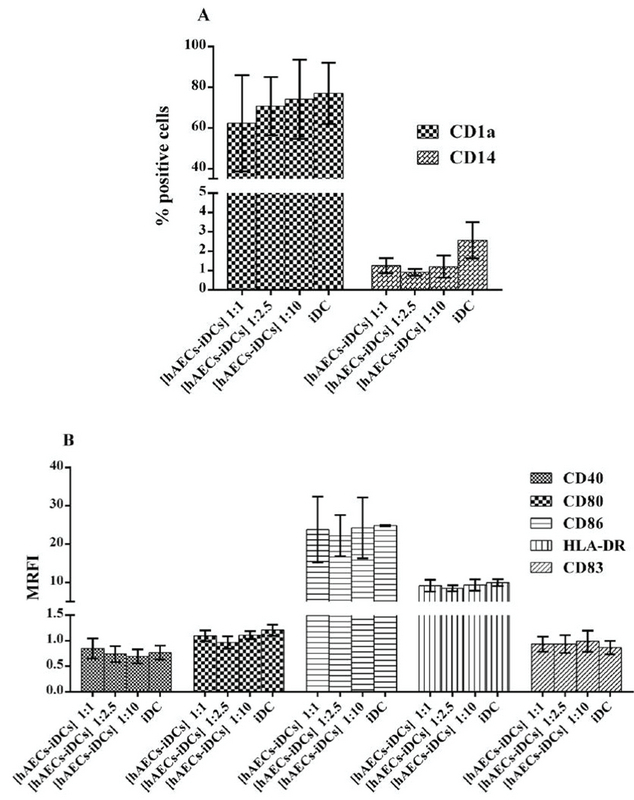
Immunophenotyping of monocyte-derived immature dendritic cells in the presence and absence of Human amniotic epithelial cells. Monocytes cultured with GM-CSF plus IL-4 for three-five days in the presence of Human amniotic epithelial cells in Transwell. Monocyte cells were cultured at ratios 1:1, 1:2.5, and 1:10 (monocytes: hAECs). [hAECs-iDCs] were analyzed for markers shown in the figure using flow cytometry. (A) The percentages of cells staining positive for CD1a and CD14. (B) The mean of Mean Ratio Fluorescence Intensity for each marker. Data are expressed as mean ± SD of four separate experiments. [hAECs-iDCs]: monocyte derived immature dendritic cells in the presence of Human amniotic epithelial cells. MRFI: Mean Ratio Fluorescence Intensity. iDC: monocyte derived immature dendritic cells in the absence of Human amniotic epithelial cells.

Monocytes co-cultured with hAEC for five days in the presence of IL-4 and GM-CSF were stimulated to differentiate into iDC cells. The cells from this co-culture were named [hAECs-iDCs]. Immunophenotyping of [hAECs-iDCs] cells revealed that the percentage of CD1a (p = 0.59) and CD14 (P-value = 0.27) cells in all three ratios of 1:1, 1:2.5, and 1:10 showed no significant difference as compared with the control group (Figure 3A). The expression of CD80 (p = 0.82), CD86 (p = 0.82), CD40 (p = 0.76), HLA-DR (p = 0.87), and CD83 (p = 0.72) markers also showed no significant difference with the control group (Figure 3B). The Kruskal-Wallis test was used to analyze groups. P < 0.05 was considered significant.

### Immunophenotyping of monocyte derived mDC cells in the presence of hAEC

**Figure 4:**
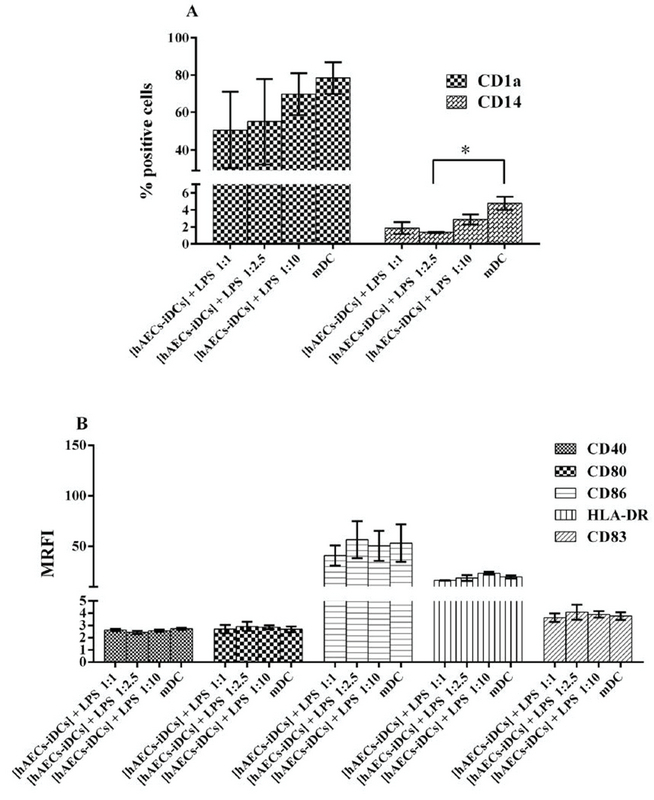
Immunophenotyping of monocyte-derived mature dendritic cells in the presence and absence of Human amniotic epithelial cells. Monocytes were cultured with GM-CSF plus IL-4 for three-five days in the presence of human amniotic membrane in Transwell. Then, immature dendritic cells were stimulated by adding 100 ng/ml LPS and incubated for two more days (day 7). The cells from this co-culture were named [hAECs-iDCs] + LPS. Monocyte cells were cultured at ratios 1:1, 1:2.5, and 1:10 (monocytes: human amniotic membrane). [hAECs-iDCs] + LPS were analyzed for markers shown in the figure using flow cytometry. (A) The percentages of cells staining positive for CD1a and CD14. (B) The mean of Mean Ratio Fluorescence Intensity for each marker. Data are expressed as mean ± SD of four separate experiments. The Kruskal-Wallis test was used to analyze. MRFI: Mean Ratio Fluorescence Intensity. mDC: monocyte derived immature dendritic cells in the absence of Human amniotic epithelial cells and then maturated with LPS for two days.

With the addition of LPS to hAECs-iDCs cells and incubation for two more days, cells were stimulated to differentiate into mDC. The cells from this co-culture were characterized as [hAECs-iDCs] + LPS cells. Immunophenotyping of [hAECs-iDCs] + LPS cells revealed that the percentage of CD1a cells in all three ratios of 1:1, 1:2.5, and 1:10 showed no significant difference as compared with the control group (p = 0.2). The Percentage of CD14 cells in the ratio 1:2.5 showed significant differences compared with the control group (p = 0.02) (Figure 4A). The expression of CD80 (p = 0.83), CD86 (p = 0.97), CD40 (p = 0.47), HLA-DR (p = 0.10), and CD83 (p = 0.93) markers also showed no significant difference with the control group (Figure 4B). The Kruskal-Wallis test was used to analyze groups. P-value < 0/05 was considered significant.

### Effect of hAECs on DC cytokine production

**Figure 5:**
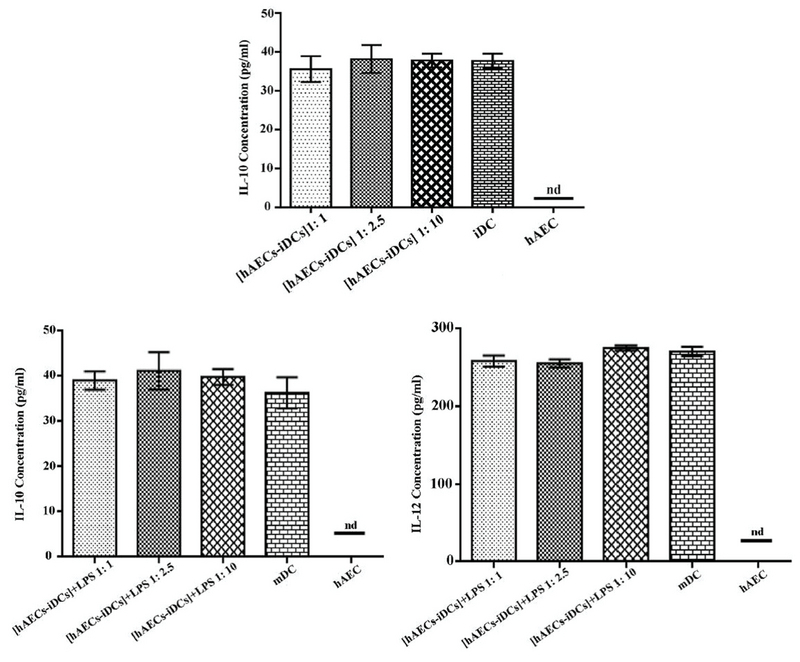
The cytokines of IL-10 and IL-12 were evaluated from supernatants of monocyte cultures differentiated toward dendritic cells in the absence (mature dendritic cells and immature dendritic cells) or presence ([hAECs-iDCs] and [hAECs-iDCs] + LPS of human amniotic epithelial cells in Transwell conditions. IL-12 was not detected in the supernatant of immature dendritic cells (data not shown). The results are the mean ± SD of four individual supernatants for each cell population. nd, not detected; hAEC, human amniotic epithelial cells; mDC, mature dendritic cells; iDC, immature dendritic cells; [hAECs-iDCs]: monocyte-derived iDC cells in the presence of hAEC; [hAECs-iDCs] + LPS, monocyte-derived iDC cells in the presence of hAEC and then maturated with LPS for two days.

Since the cytokine microenvironment has a very important role in the maturation, recruitment, and functioning of DCs, we next evaluated IL-10 and IL-12 in supernatant of co-cultures in all three ratios of 1:1, 1:2.5, and 1:10. As shown in Figure 5, in production of IL-10 and IL-12 there is no significant difference in any of the cultures as compared to the control groups. We used one-way analysis of variance (ANOVA) test to analyze groups. IL-12 was not detected in the supernatant of iDC cells (data not shown).

### hAECs don't affect the ability of DC to induce PBL proliferation 

**Figure 6:**
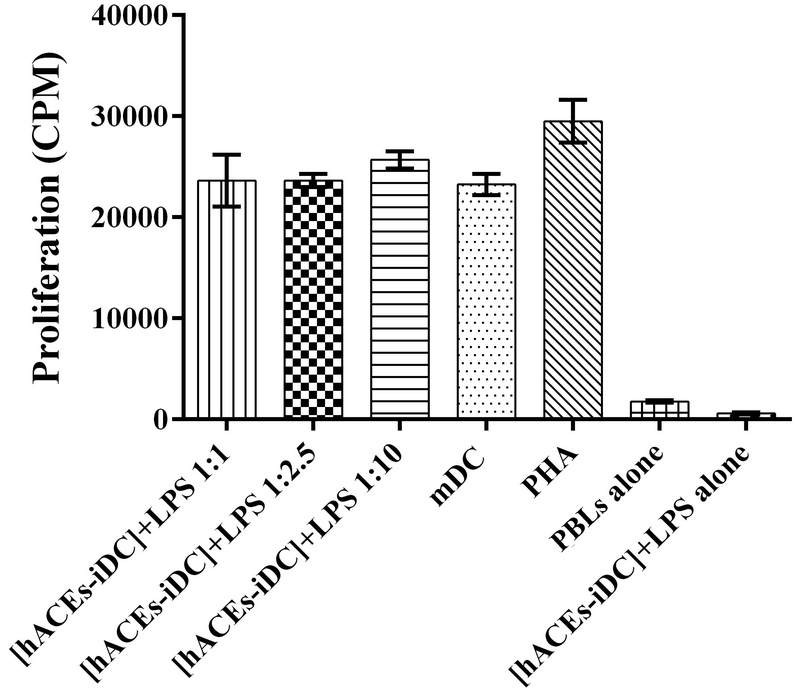
Effect of hAECs-treated monocyte-derived mDCs on PBL cells proliferation. Mature dendritic cells (control group) and [hAECs-iDCs] + LPS were collected after culture and used as stimulators to induce proliferation of peripheral blood lymphocytes cells (responders). Co-culture of [hAECs-iDCs] + LPS cells with peripheral blood lymphocytes cells were used as test, co-culture of mDC with peripheral blood lymphocytes cells were used as control, [hAECs-iDCs] + LPS cells and peripheral blood lymphocytes cells alone were used as negative controls, co-culture of Phytohemagglutinin with peripheral blood lymphocytes cells was used as positive control. T cell proliferation was measured by [3H] thymidine incorporation assessment after five days of culture and expressed as counts per minute (cpm). Average cpm for [hAECs-iDCs] + LPS cells in the three ratios of 1:1, 1:2.5, and 1:10 was 23630 ± 644/8, 23608 ± 846/4, and 25674 ± 2560, respectively, and for mature dendritic cells 23237 ± 1047. The results shown are the mean ± SD of four individual experiments. hAEC, human amniotic epithelial cells; mDC, mature dendritic cells; PBL, Peripheral blood lymphocytes; PHA, Phytohemagglutinin; [hAECs-iDCs] + LPS, monocyte derived immature dendritic cells in the presence of hAEC and then maturated with LPS for two days.

To evaluate the functional ability of [hAECs-iDCs] + LPS cells to induce the PBL cells proliferation, MLR test was used. According to the results, there was no significant difference between [hAECs-iDCs] + LPS and mDC cells in stimulation of PBLs cells proliferation. Average CPM for [hAECs-iDCs] + LPS cells in the three ratios of 1:1, 1:2.5, and 1:10 was 23630 ± 644/8, 23608 ± 846/4, and 25674 ± 2560, respectively, and for mDC 23237 ± 1047 (Figure 6). We used one-way ANOVA test to analyze groups.

### hAECs don't affect the ability of DC to induce Treg expansion

**Figure 7:**
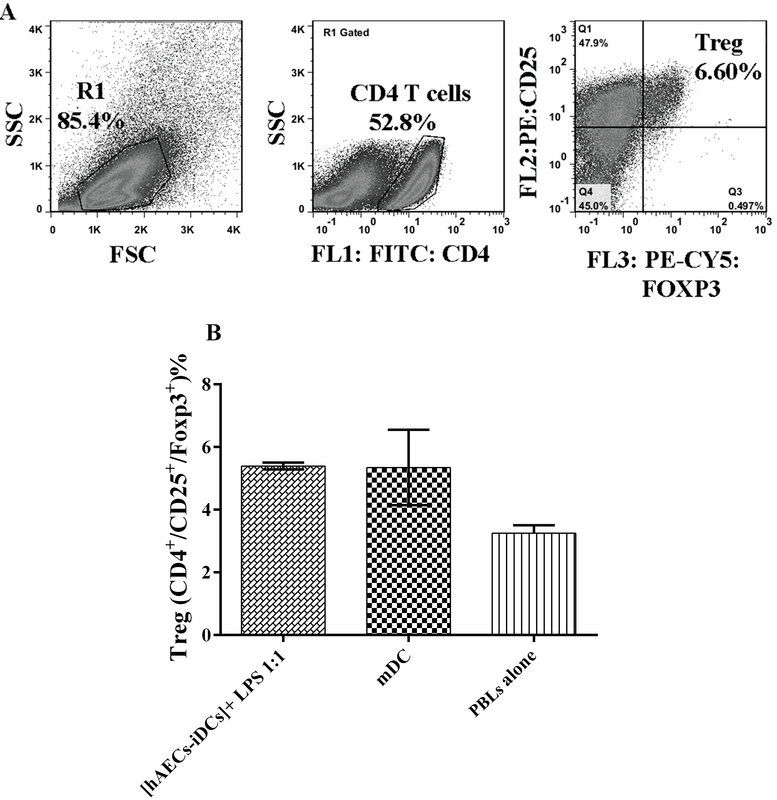
Regulatory T cells assay. In order to evaluate the [hAECs-iDCs] + LPS cells' ability to generate T-reg cells, [hAECs-iDCs] + LPS and mature dendritic cells were co-cultured with peripheral blood lymphocytes cells for five days at the ratio 1:1. Peripheral blood lymphocytes cells alone were used as negative controls. Cells were stained by Human Regulatory T Cell Staining Kit and anti-CD4, CD25, and Foxp3 antibodies and analyzed by flow cytometry. (A) indicates the flow cytometry gating strategy used to quantitate CD4 + CD25 + FoxP3 + Treg cells. As shown in (B), in the percentage of Treg cells there was no significant difference between [hAECs-iDCs] + LPS and mature dendritic cells. mDC, mature dendritic cells; PBL, Peripheral blood lymphocytes; [hAECs-iDCs] + LPS, monocyte-derived iDC cells in the presence of hAEC and then maturated with LPS for two days.

In order to assess the [hAECs-iDCs]+ LPS cells' ability to induce T-reg cells, [hAECs-iDCs]+ LPS and mDC cells were co-cultured with PBL cells for five days at the ratio 1:1. PBL cells alone were used as negative controls. According to the results, in MLR reaction, the percentage of Treg cells did not show significant difference between [hAECs-iDCs] + LPS (and mDC cells (Figure 7) (P-value = 0.99). One-way analysis Mann-Whitney test was used to analyze groups. P-value < 0/05 was considered significant.

## 4. Discussion

Based on the results, although hAECs could not significantly affect the differentiation of monocytes into DCs in our experimental settings, but they were able to partially decrease the expression of CD1a and CD14 at co-culture ratios of 1:1 and 1:2.5, respectively. There are many conflicting studies regarding the immunomodulatory properties of hAECs' secreted factors.

Our team has successfully isolated and characterized hAECs in a previous study (22). Based on the findings, harvested hAECs possessed many of the MSC properties (22). Keeping in mind the known immunosuppressive function of these cells during pregnancy, we have evaluated in the present study the immunomodulatory properties of soluble factors secreted from hAECs on DC differentiation and cytokine production taking advantage of a Transwell co-culture system.

DCs, as the central regulators of the immune system, are among the most important cells affected by such an environment, which can largely influence the fate of the pregnancy (14). It is also known that the amniotic membrane is a key role player in the maintenance of human pregnancy, which consists of two major cell types including hAMCs and hAECs. In this regard, hAMCs have been shown by several studies to exert immunomodulatory effects on DC maturation in both indirect (Transwell) and direct contact co-culture systems (17, 23). Acknowledging the findings of previous studies indicating that hAECs share some of the immunomodulatory properties with hAMCs (2, 10), we made an effort in the present study to check whether the hAECs isolated from human amniotic membrane during Cesarean sections of women with term pregnancies can also affect the differentiation of monocytes toward DCs.

Some studies have demonstrated that B and T cells proliferation as well as the chemotactic activities of neutrophils and macrophages as evidenced by the production of the macrophage inflammatory protein 2 (MIP)-2 can be inhibited by the supernatant from hAECs cultures (2). While other studies have shown that direct cell-to-cell contact is necessary for the AECs to exert their immunomodulatory effects (10, 17). In agreement with these findings, Magatti and colleagues showed that the effect of hAECs on monocytes differentiation toward DCs and the ability of these differentiated DCs to induce T cell proliferation were more pronounced in the cell-to-cell contact co-culture system compared to the indirect Transwell system (17). These findings all together show that although the soluble factors secreted from hAECs possessed immunomodulatory properties and could inhibit the proliferation of immune cells in many studies, these soluble factors alone, however, were unable both effectively influence the differentiation of monocytes toward effector DCs and to convert DCs to tolerogenic phenotypes. In contrast, signals received from hAECs during cell-to-cell crosstalk could effectively promote differentiation of monocytes toward tolerogenic DCs. The mechanisms responsible for these events are still not completely understood. Studying the ability of cell-to-cell contact alone in the absence of hAECs-secreted factors to induce tolerogenic DCs remain unanswered because of the limitations in the design of such an experimental model.

In another study, Magatti and colleagues observed that in contrast to hAMCs, the inhibitory capacity of hAECs correlated inversely with their numbers in the culture. These findings reveal that the determination of an optimal dose is an essential prerequisite for the clinical and experimental application of hAECs. Many studies have indicated that increasing the passage number of hAECs leads to the acquisition of mesenchymal markers (such as CD105, CD13, CD44, and CD90) as well as epithelial-mesenchymal transition (24-26). Such a phonotypic shift led to a reduced immunomodulatory capacity of hAECs in these studies (17). Considering these important findings, we used hAECs at passage 0 in our experiments to avoid such a deteriorating phenotypic change. It is well-known that the cytokine profile, the ability to stimulate T cells, and the functional capacity of DCs are the three important arms implicated in promoting the immune response (27). Therefore, in the present work, we evaluated two cytokines, that is, IL-10 and IL-12 in supernatant of the co-cultures. Neither the production of IL-10 nor of IL-12 differed significantly between the test groups and the control group, which is consistent with the findings of the study by Magatti and colleagues in which the inhibitory effects of hAECs on the production of inflammatory cytokines were only seen in the direct cell-to-cell contact co-culture system (17). According to another study by Chang and colleagues (28), the mDC type 1, which is the same DC produced in our study, produces very small amounts of IL10; which is in parallel with our findings. Our findings on the functional ability of generated DC (in the presence of hAECs) to induce PBL proliferation and Treg expansion in MLR reaction indicated that PBLs co-cultured with hAECs. Moreover, we observed that hAECs don't affect cytokine production and Treg expansion of DCs. In line with this, Magatti and colleagues indicated that only DCs generated in the direct cell-to-cell system but not those generated in the Transwell system had a significantly reduced ability to stimulate T-cell proliferation. Moreover, the cytokine profile of hAEC-treated DCs was more dominant in the direct cell-to-cell contact condition, compared to the Transwell system (17). All in all, these findings along with the findings of previous studies are in agreement with our results emphasizing the inability of hAECs soluble factors to influence monocyte differentiation into DCs as well as the necessity of a direct cell-to-cell contact for the hAECs-driven immunomodulatory effects to take place.

## 5. Conclusion 

Our findings show that the factor secreted from freshly isolated hAEC at the primary passage (passage 0) is unable to inhibit the generation of mDCs. Therefore, perhaps the immunomodulatory effects of hAECs in diseases of inflammatory or autoimmune pathogenesis is mediated through a direct cell-to-cell contact mechanism. Indeed, further research with proper experimental designs will aid in uncovering the ambiguous aspects of these findings.

##  Conflict of Interest 

The authors have no competing interests to report.
